# The association between animal protein, plant protein, and their substitution with bladder cancer risk: a pooled analysis of 10 cohort studies

**DOI:** 10.1007/s00394-024-03551-3

**Published:** 2024-12-24

**Authors:** Sara Beigrezaei, Mostafa Dianati, Amin Salehi-Abargouei, Mohammad Fararouei, Ali Akbari-Beni, Maree Brinkman, Emily White, Elisabete Weiderpass, Florence Le Calvez-Kelm, Marc J. Gunter, Inge Huybrechts, Fredrik Liedberg, Guri Skeie, Anne Tjonneland, Elio Riboli, Maurice P. Zeegers, Anke Wesselius

**Affiliations:** 1 Department of Global Public Health and Bioethics, Julius Center for Health Sciences and Primary Care, University Medical Center (UMC) Utrecht, Utrecht, The Netherlands; 2 International Agency for Research on Cancer World Health Organization, Lyon, France; 3 School of Medical and Life Sciences, Sunway University, Sunway City, Malaysia; 4 Nutrition and Food Security Research Center, Department of Nutrition, School of Public Health, Shahid Sadoughi University of Medical Sciences, Yazd, Iran; 5 Department of Epidemiology, Shiraz University of Medical Sciences, Shiraz, Iran; 6 Department of Cell & Molecular Biology, University of Mississippi Medical Center, 2500 North State Street, Jackson, MS 39216 USA; 7 Department of Epidemiology, Maastricht University, Universiteitssingel 40 (Room C5.570), Maastricht, 6229 ER The Netherlands; 8 Department of Clinical Studies and Nutritional Epidemiology, Nutrition Biomed Research Institute, Melbourne, Australia; 9 Cancer Epidemiology Division, Cancer Council Victoria, 615 St Kilda Road, Melbourne, VIC 3004 Australia; 10 Fred Hutchinson Cancer Research Center, Seattle, WA USA; 11 Department of Urology, Skåne University Hospital, Malmö, Sweden; 12 Institution of Translational Medicine, Lund University, Malmö, Sweden; 13 Department of Community Medicine, UIT The Arctic University of Norway, Tromsø, Norway; 14 Danish Cancer Society Research Center, Copenhagen, Denmark; 15 Department of Public Health, University of Copenhagen, Copenhagen, Denmark; 16 Department of Epidemiology and Biostatistics, School of Public Health, Imperial College London, London, UK; 17 CAPHRI School for Public Health and Primary Care, Maastricht University, Maastricht, The Netherlands; 18 School of Cancer Sciences, University of Birmingham, Birmingham, UK

**Keywords:** Total proteins, Plant-based proteins, Animal-based proteins, Bladder cancer, Substitution analysis, Replacement, Cohort studies

## Abstract

**Purpose:**

Although total dietary protein intake has been associated with bladder cancer (BC) risk, the effect of the origin (plant or animal) and the substitutions remain to be understood. This study aimed to investigate the effect of total dietary protein, animal-based protein, plant-based protein, and their substitutions with each other on the risk of BC using a pooled analysis of 10 cohort studies.

**Methods:**

The study was conducted within the “BLadder cancer Epidemiology and Nutritional Determinants” (BLEND) study, including 10 prospective cohort studies from several European countries, the United Kingdom, and the United States. Individual data from 10 prospective cohorts containing 434,412 participants (overall male/female ratio was almost 3:1) with a total of 4,224,643.8 person-years of follow-up was analyzed. Hazard ratios (HRs) and 95% confidence intervals (CIs) for BC risk for animal and plant-based protein substitutions of 30gram (g) per day (g/day) were estimated by multivariable adjusted HRs using Cox proportional hazards models.

**Results:**

During 11.4 years of follow-up, among 434,412 participants (73.28% female), 1,440 new cases of BC were identified. After multivariable adjustment, no association was observed between the intake of total, animal-based protein, and plant-based protein and BC risk. Replacement of every 30 g/day of animal-based protein intake by the same amount of plant-based protein intake or vice versa was not associated with the risk of BC.

**Conclusion:**

In conclusion, our study found no association between protein intake—whether from animal or plant sources—and the risk of BC. Substituting animal-based protein with plant-based protein, or the reverse, did not influence BC risk. Future studies are required to provide information on the link between animal- and plant-based proteins and BC risk.

**Supplementary Information:**

The online version contains supplementary material available at 10.1007/s00394-024-03551-3.

## Introduction

Bladder cancer (BC) is the 10th most common and 13th most deadly neoplasm worldwide, and the number of incident cases and deaths related to the condition is still rising [1, 2], particularly in Europe and other developed nations. The incidence rate of BC and its mortality is approximately four times higher in men than in women [2]. Due to the high recurrence rate of BC and the ongoing invasive monitoring requirement, BC has the highest per patient lifetime treatment costs of all cancers, posing a remarkable financial burden on the healthcare systems [3, 4]. New preventive and management strategies are, therefore, highly needed [5]. Previous epidemiologic research has demonstrated that most BC cases are attributable to tobacco use, male sex, age, obesity, and occupational exposures [6–8]. In addition, as for many cancers, the association of various dietary factors with BC has been studied [9–11], and the results were supportive of the benefits of consuming more vegetables and fruits [12], dairy products [13], and tea [14]. However, despite the currently available evidence on the association between diet and BC risk, a recent report by the World Cancer Research Fund International (WCRF) declared that the evidence is still scarce [15].

Dietary protein intake has been a high-priority research topic of interest in nutritional research [16]. It has been demonstrated that even small changes in the amount or combination of protein intake in individuals can have a big impact on public health [17, 18]. Evidence from prospective cohort studies has demonstrated that different sources of dietary protein might have the potential to affect the development of several chronic diseases [19, 20]. There are also some previous studies on different sources of protein and BC risk [21, 22]. Several prospective cohort studies have shown an increased risk of BC associated with increased consumption of red and processed meat [21, 23]. In contrast, Dianatinasab et al. [24] reported an elevated risk of BC specifically linked to high organ meat intake, while no significant associations were observed for other meat sources. Similarly, a Swedish cohort study found no significant association between the intake of red and processed meat, poultry, or fish and BC risk [22]. Despite prior research on the associations between the intake of different protein sources and BC risk, the overall results remain inconclusive. Dietary proteins are macronutrients that are classified as animal and plant origins [25]. Proteins originating from animal or plant sources have different combinations of amino acids and dietary compounds with potentially various health effects [26].

In nutritional epidemiology, dietary substitution methods are used to evaluate the impact of replacing specific foods with other foods of equivalent amounts on the risk of disease development [27]. Substitution analyses provide a statistical framework that models dietary modifications by altering macronutrient composition while maintaining constant total energy intake, thereby helping to identify optimal dietary patterns.

To our best knowledge, previous studies have not focused on the effect of different origins of dietary protein and their replacement on BC risk and data on the theoretical effects of substituting protein sources on the risk of BC is limited. Therefore, the present study aimed to investigate the effect of total, animal, and plant-based proteins, as well as their replacement, on the risk of developing BC, by using the pooled data of 10 cohort studies.

## Materials and methods

### Study population

Data for this investigation was obtained from the Bladder Cancer Epidemiology and Nutritional Determinants Consortium (BLEND) [28]. The BLEND study is a large international consortium that merges individual participant data from 19 case-control studies and 16 cohort studies [28]. For the present study, data from 10 cohort studies, with a total of 434,412 participants (1440 BC cases and 432,972 non-cases), from centers in 10 countries [i.e. Europe: European Prospective Investigation into Cancer and Nutrition cohort studies (EPIC) [29] (Denmark [30], France [31], Germany [32], Italy [33], The Netherlands [34], Norway [35], Spain [36], Sweden [37, 38], United Kingdom [39, 40]); and North America: (USA) (Vitamins and Lifestyle cohort study (VITAL)) [41]] were included (Supplementary Table 1).

Studies were selected based on the availability of comprehensive data on all relevant variables, including dietary intake and BC incidence. Studies with missing information on dietary intake or incomplete data on BC diagnosis were excluded to ensure the accuracy and robustness of the results.

### Data collection and coding

Details on the protocol and methodology of the BLEND consortium have been described elsewhere [28]. In brief, the primary data from the included studies were collected into an integrated database. All data were checked, and all food intakes were converted to an intake of grams per day (g/day) using country-specific food tables and the frequency responses in the dietary questionnaires. All the included studies identified new cases of BC by including participants diagnosed with urinary bladder neoplasms, as defined by the International Classification of Diseases for Oncology (ICD-O-3 code C67). These diagnoses were determined through self-reported questionnaires, population-based cancer registries, health insurance records, or medical records [42, 43].

The collected dietary data was harmonized and categorized by using the hierarchical Eurocode 2 food coding system developed by the European Union [44], besides, weekly, monthly or yearly intake were converted to weekly food intake. Dietary intake data were obtained using validated semi-quantitative food frequency questionnaires (FFQs) [41, 45–47] and recorded using the Eurocode 2 food coding system [48]. Total dietary protein intake was categorized into two macronutrient groups: (a) animal protein (derived from animal-based foods such as meats, fish, milk, and eggs) and (b) plant protein (derived from plant-based foods such as fruits, vegetables, legumes, grains, nuts, and beverages).

The intake of these dietary proteins was calculated in grams per 1000 kilocalories per day (g/1000 kcal/day, nutrient density method), to account for total energy intake and reduce extraneous variation in dietary intakes [49], and were categorized into tertiles (i.e. low intake (tertile 1), medium intake (tertile 2), and high intake (tertile 3).

Alcohol intake was considered as the sum of dietary intake of beer (g/day) and wine (g/day). In addition to dietary intake data, the data on age, sex, smoking status (current/former/never), and smoking pack-years measured at baseline using a demographic questionnaire.

### Statistical analyses

The differences between the baseline characteristics of the participants based on tertiles of dietary total protein intake were assessed by the ANOVA test for continuous variables and the Chi-square for categorical variables. All statistical analyses were performed on the pooled data from the 10 cohort studies included in our analysis.

Cox proportional hazard models using age as the underlying time metric were used to assess the association between protein intake and BC risk. Hazard ratios (HRs) and 95% confidence intervals (CIs) were calculated using the first tertile as the reference group. The proportional hazard assumption was graphically examined, and no evidence of violation was found. The follow-up time was calculated from the date of study enrolment and the date of a registered diagnosis of BC or the date of last follow-up (date of death, lost to follow-up, or study exit). The Cox regression models were performed as Model 1 (adjusted based on total energy intake (kcal)), Model 2 (adjusted based on Model 1 and additionally adjusted for age and sex), and Model 3 (adjusted based on Model 2 and additionally for smoking status (never, former, or current smoker) and alcohol intake (g/day). Total dietary protein intake was divided into wo macronutrient groups: animal protein and plant protein. Animal protein, derived from animal-based food groups including red meat, white meat, fish, dairy products, and eggs. Red meat included all forms of fresh (e.g., beef, pork, hamburger, liver, and meats used in dishes) and processed red meat (e.g., bacon, ham, and sausage). White meat encompassed chicken, turkey, and other white meats. Fish, including fish, canned tuna, and other sea foods and dairy products including milk, cheese, yoghurt, and other dairy products. Plant protein, derived from plant-based food groups including bread, cereal, pasta, nuts, beans, legumes, beverages, fruits, and vegetables. To assess the effect of substituting animal-based proteins with plant-based proteins and vice versa, we used a leave-one-out model [27]. For this, 30 g/day [50] of animal-based proteins was replaced by the same quantity of plant-based proteins, and vice versa, while keeping the total energy intake constant. This means that an increase in plant-based protein was offset by an equal decrease in animal-based protein, ensuring that both total protein and energy intake remains constant. Similarly, an increase in animal-based protein was offset by an equal decrease in plant protein, with total protein and energy intake remains constant. In addition, we examined the non-linear dose-response association between the intake of animal-based protein to total protein ratio (%) and BC risk using a restricted cubic spline model with a fully adjusted model. The P for heterogeneity was calculated using the Wald test. Four knots were selected at the following animal to total protein ratio percentages: 0%, 5%, 10%, 20%, 30%, 40%, 50%, 60%, 70%, 80%, 90%, and 100%. The reference value was fixed at an animal to total protein ratio of less than 5% of total energy intake from protein.

Secondary analyses included the evaluation of a potential interaction between protein intake and sex (the interaction effect was checked in the main model) and omitting BC cases diagnosed in the first two years of follow-up. Finally, to determine the single study effect, sensitivity analyses were performed by removing each individual study in turn from the main analysis. The data was analyzed using Stata statistical software (version 14.2). P-values less than 0.05 were considered statistically significant.

## Results

### Baseline characteristics

The baseline characteristics of the study population across tertiles of the total protein intake are provided in Table 1. In total, 434,412 participants (318,343 women and 116,069 men), including 1,440 incident BC cases, with a total of 4,224,643.8 person-years were included. The median follow-up was 11.4 [range = 0.003, 31.34] years. Participants in the highest tertile of total protein intake were more likely to be current smokers than those in the lowest tertile (Table 1).

**Table 1:** Baseline characteristics of participants based on tertiles of total protein intake

Variables	All participants	Total protein intake			*P*-value
		Tertile 1 < 28.4 g/day	Tertile 2 28.4–40.8 g/day	Tertile 3 > 40.8 g/day	
Age at baseline (year) [mean ± SD]	50.6 ± 10.2	49.9 ± 11.1	50.3 ± 10.3	51.5 ± 9.0	< 0.001*
Sex					< 0.001^†^
Women, n (%)	318,343 (73.3)	114,048 (78.3)	106,790(73.7)	98,232 (67.8)	
Men, n (%)	116,069 (26.7)	31,483 (21.7)	38,014 (26.3)	46,572 (32.2)	
Smoking status					< 0.001^†^
Current smoker, n (%)	92,045 (21.2)	28,243 (19.5)	31,573 (21.8)	32,229 (22.3)	
Former smoker, n (%)	129,547 (29.8)	45,496 (31.4)	43,881 (30.3)	40,170 (27.7)	
Never smoker, n (%)	212,820 (49)	71,065 (49.1)	69,350 (47.9)	72,405 (50.0)	
Energy intake, kcal/day	2160.5 ± 664.4	1861.1 ± 542.1	2100.5 ± 569.6	2481.3 ± 706.3	< 0.001*
Total protein intake, g/day	38.1 ± 17.4	22.5 ± 4.1	34.1 ± 3.5	57.5 ± 15.7	< 0.001*
Total protein intake, energy %	16.5 ± 4.1	15.0 ± 3.4	17.2 ± 3.6	17.3 ± 4.8	< 0.001*
Animal-based protein intake, g/day	24.5 ± 14.1	14.9 ± 6.1	22.3 ± 8.4	36.2 ± 15.9	< 0.001*
Animal-based protein intake, energy %	10.5 ± 4.1	9.8 ± 3.9	10.9 ± 3.8	10.8 ± 4.2	< 0.001*
Plant-based protein intake, g/day	13.6 ± 12.2	6.8 ± 5.2	11.2 ± 7.7	21.6 ± 15.7	< 0.001*
Plant-based protein intake, energy %	5.6 ± 4.5	4.6 ± 3.8	5.9 ± 4.8	6.3 ± 4.5	< 0.001*
Alcohol intake, g/day	9.4 ± 15.0	6.9 ± 11.6	8.89 ± 14.46	12.3 ± 17.7	< 0.001*

Data are presented as mean ± SD for quantitative variables and as number, (%) for qualitative variables

*Based on independent sample t-test

^†^Based on Chi-2 test

Standard deviation, SD; g, gram; kcal, kilocalorie

### Total dietary protein intake and BC risk

Results of the association between total dietary protein intake and BC risk are provided in Table 2. The linear model showed that an additional 30 g/day of total protein intake was not related to the risk of BC. The linear model in women showed that an additional 30 g/day of total protein intake was associated with an overall 40% decrease in the risk of BC after adjustment for age, sex, and energy intake (Model 2: HR:0.60; 95%CI: 0.47, 0.77), however this association disappeared after additional adjustments for smoking status and alcohol intake in final model. No significant association was observed in men.

**Table 2:** The association between dietary intakes of total protein and bladder cancer

	Linear association (per 30 g)	Nonlinear association			
		Tertile 1 < 28.4 g/day	Tertile 2 28.4–40.8 g/day	Tertile 3 > 40.8 g/day	*P* trend
	HR (95% CI)	HR (95% CI)	HR (95% CI)	HR (95% CI)	
**All participants** (cases/non-cases)	1,440/432,972	459/144,345	476/144,328	505/144,299	
Person-year	4,224,643.8	1,324,858.2	1,395,118.8	1,504,666.8	
Model 1	0.95 (0.84, 1.08)	1 (ref.)	1.11 (0.97, 1.26)	1.13 (0.96, 1.32)	0.09
Model 2	0.95 (0.81, 1.11)	1 (ref.)	1.03 (0.90, 1.18)	1.01 (0.86, 1.18)	0.98
Model 3	0.95 (0.81, 1.05)	1 (ref.)	1.01 (0.88, 1.17)	1.01 (0.85, 1.20)	0.88
**Based on sex** (P for interaction > 0.05)					
**Men** (cases/non-cases)	986/115,083	281/31,202	319/37,695	386/46,186	
Model 1	1.14 (0.96, 1.34)	1 (ref.)	1.07 (0.91, 1.26)	1.20 (1.00, 1.45)	0.05
Model 2	1.14 (0.97, 1.35)	1 (ref.)	1.07 (0.92, 1.26)	1.21 (1.00, 1.46)	0.05
Model 3	1.04 (0.86, 1.25)	1 (ref.)	1.04 (0.87, 1.25)	1.10 (0.89, 1.35)	0.36
**Women** (cases/non-cases)	454/317,889	178/113,143	157/106,633	119/98,113	
Model 1	0.59 (0.46, 0.76)	1 (ref.)	0.96 (0.76, 1.21)	0.63 (0.46, 0.86)	0.01
Model 2	0.60 (0.47, 0.77)	1 (ref.)	0.97 (0.77, 1.21)	0.64 (0.47, 0.87)	0.01
Model 3	0.78 (0.57, 1.05)	1 (ref.)	0.96 (0.75, 1.22)	0.81 (0.58, 1.13)	0.26

HR, hazard ratio; CI, confidence interval; g, gram

Model 1: Adjusted based on total energy intake

Model 2: Additionally, adjusted based on age and sex

Model 3: Additionally, adjusted based on smoking status, and alcohol intake

The non-linear model showed that higher total protein intake was associated with a lower BC risk among women after adjustment for age, sex, and energy intake (Model 2: HR_high vs. low_:0.64; 95%CI: 0.47, 0.87, p-trend = 0.01), however no association was observed in the final adjusted model (Table 2).

### Animal dietary protein intake and BC risk

Overall, the linear model revealed a direct significant association between every 30 g/day increase in animal-based protein intake and the risk of BC after adjusting for age, sex, and energy intake (Model 2: HR:1.23; 95%CI: 1.05, 1.44) (Table 3). Stratification based on sex showed the same results only among men. These associations became non-significant in final adjusted model.

**Table 3:** The association between dietary intakes of animal-based protein and bladder cancer

	Linear association (per 30 g)	Nonlinear association (per 30 g)			
		Tertile 1 < 6.6 g/day	Tertile 2 6.6–15.2 g/day	Tertile 3 > 15.2 g/day	*P* trend
	HR (95% CI)	HR (95% CI)	HR (95% CI)	HR (95% CI)	
**All participants** (cases/non-cases)	1,440/432,972	370/145,273	546/144,888	524/142,811	
Person-year	4,224,643.8	1,544,886.6	1,424,251.8	1,255,505.4	
Model 1	1.65 (1.42, 1.93)	1 (ref.)	1.31 (1.14, 1.50)	1.41 (1.23, 1.63)	< 0.001
Model 2	1.23 (1.05, 1.44)	1 (ref.)	1.21 (1.06, 1.38)	1.14 (0.99, 1.32)	0.06
Model 3	1.12(0.92, 1.38)	1 (ref.)	1.13 (0.98, 1.30)	1.05 (0.90, 1.24)	0.44
**Based on sex** (P for interaction > 0.05)					
**Men** (cases/non-cases)	986/115,083	237/34,006	357/38,558	392/42,519	
Model 1	1.24 (1.04, 1.49)	1 (ref.)	1.20 (1.02, 1.42)	1.20 (1.01, 1.43)	0.04
Model 2	1.24 (1.03, 1.48)	1 (ref.)	1.18 (1.00, 1.39)	1.19 (1.00, 1.42)	0.05
Model 3	1.16 (0.92, 1.46)	1 (ref.)	1.09 (0.92, 1.30)	1.09 (0.90, 1.32)	0.37
**Women** (cases /non-cases)	454/317,889	133/111,267	189/106,330	132/100,292	
Model 1	1.14 (0.81, 1.62)	1 (ref.)	1.26 (1.01, 1.59)	1.00 (0.76, 1.32)	0.77
Model 2	1.16 (0.82, 1.64)	1 (ref.)	1.25 (0.99, 1.57)	0.99 (0.75, 1.31)	0.83
Model 3	0.98 (0.63, 1.52)	1 (ref.)	1.20 (0.94, 1.53)	0.94 (0.68, 1.28)	0.99

HR, hazard ratio; CI, confidence interval; g, gram

Model 1: Adjusted based on total energy intake

Model 2: Additionally, adjusted based on age and sex

Model 3: Additionally, adjusted based on smoking status and alcohol intake

The modest intake of animal-based protein intake showed a higher risk of BC in Model 2 (Model 2: HR_second tertile vs. lowest_: 1.21; 95%CI: 1.06, 1.38, p-trend = 0.06), however no association remained in the final adjusted model (Table 3).

### Plant-based protein intake and BC risk

The linear model showed that an additional 30 g/day of plant-based protein intake was associated with 34% decreased risk of BC after adjustment for age, sex, and energy intake (Model 2: HR:0.76; 95%CI: 0.65, 0.88) (Table 4). Moreover, an additional 30 g/day of plant-based protein intake was associated with lower risk of BC in Model 2: HR:0.51; 95%CI: 0.38, 0.67, however these associations disappeared after additional adjustments for smoking status and alcohol intake in the final adjusted model. There was no significant association between plant-based protein intake and BC risk among men (Table 4).

**Table 4:** The association between dietary intakes of plant-based proteins and bladder cancer

	Linear association (per 30 g)	Nonlinear association (per 30 g)			
		Tertile1 < 17.9 g/day	Tertile2 17.9–28.0 g/day	Tertile3 > 28.0 g/day	*P* trend
	HR (95% CI)	HR (95% CI)	HR (95% CI)	HR (95% CI)	
**All participants** (cases /non-cases)	1,440/432,972	541/144,841	497/143,893	402/144,238	
Person-year	4224838.7	1087839.3	1569512.2	1567487.2	
Model 1	0.65 (0.56, 0.75)	1 (ref.)	0.88 (0.78, 1.00)	0.74 (0.64, 0.86)	< 0.001
Model 2	0.76 (0.65, 0.88)	1 (ref.)	1.01 (0.89, 1.15)	0.81 (0.70, 0.94)	0.006
Model 3	0.86 (0.73, 1.02)	1 (ref.)	1.02 (0.88, 1.17)	0.90 (0.77, 1.06)	0.21
**Based on sex** (P for interaction > 0.05)					
**Men** (cases/non-cases)	986/115,083	360/35,793	324/36,466	302/42,824	
Model 1	0.93 (0.78, 1.11)	1 (ref.)	1.02 (0.87, 1.19)	0.92 (0.78, 1.10)	0.41
Model 2	0.94 (0.78, 1.12)	1 (ref.)	1.04 (0.89, 1.22)	0.93 (0.78, 1.11)	0.36
Model 3	0.92 (0.75, 1.12)	1 (ref.)	1.02 (0.85, 1.21)	0.92 (0.76, 1.11)	0.36
**Women** (cases/non-cases)	454/317,889	181/109,048	173/107,427	100/101,414	
Model 1	0.50 (0.38, 0.66)	1 (ref.)	0.94 (0.75, 1.18)	0.59 (0.45, 0.78)	< 0.001
Model 2	0.51 (0.38, 0.67)	1 (ref.)	0.95 (0.75, 1.19)	0.58 (0.44, 0.77)	0.01
Model 3	0.76 (0.55, 1.05)	1 (ref.)	1.02 (0.79, 1.30)	0.88 (0.65, 1.18)	0.44

HR, hazard ratio; CI, confidence interval; g, gram

Model 1: Adjusted based on total energy intake

Model 2: Additionally, adjusted based on age and sex

Model 3: Additionally, adjusted based on smoking status and alcohol intake

In the non-linear model, higher plant-based protein intake was associated with a lower BC risk in overall after adjustment for age, sex, and energy intake (Model 2: HR_high vs. low_:0.81; 95%CI: 0.70, 0.94, p-trend = 0.006) and among women (Model 2: HR_high vs. low_:0.58; 95%CI: 0.44, 0.77, p-trend = 0.01), however these significant links disappeared in the final adjusted model (Table 4).

### Substitution of protein sources and BC risk

Table 5 presents the association between the substitution of 30/day of plant-based protein with animal-based protein and vice versa, and BC risk. Replacing 30 g/day of plant-based protein with an equal amount of animal-based protein was associated with a higher BC risk after adjusting for age, sex, and energy intake (Model 2: HR: 1.37; 95% CI: 1.13, 1.67) and similar findings were observed for men (Model 2: HR: 1.75; 95% CI: 1.16, 2.65) and women (Model 2: HR: 1.78; 95% CI: 1.18, 2.70). However, these associations did not remain in the final adjusted model.

**Table 5:** Substitution models where 30 g/day of dietary protein sources with each other

		Animal-based protein for plant-based protein (30 g/day)			Plant-based protein for animal-based protein (30 g/day)		
		Model 1 h (95% CI)	Model 2 h (95% CI)	Model 3 h (95% CI)	Model 1 h (95% CI)	Model 2 h (95% CI)	Model 3 h (95% CI)
**All participants**		1.93 (1.59, 2.33)	1.37 (1.13, 1.67)	1.17 (0.92, 1.48)	0.52 (0.43, 0.62)	0.72 (0.59, 0.88)	0.85 (0.67, 1.08)
Based on sex*	**Men**	1.18 (0.95, 1.48)	1.75 (1.16, 2.65)	1.16 (0.89, 1.52)	0.84 (0.67, 1.04)	0.84 (0.67, 1.05)	0.85 (0.65, 1.11)
	**Women**	1.93 (1.30, 2.86)	1.78 (1.18, 2.70)	1.18 (0.72, 1.95)	0.56 (0.37, 0.86)	0.56 (0.37, 0.85)	0.84 (0.51, 1.38)

HR, hazard ratio; CI, confidence interval; g, gram

Model 1: Adjusted based on total energy intake

Model 2: Additionally, adjusted based on age, sex

Model 3: Additionally, adjusted based on smoking status and alcohol intake

*P heterogeneity > 0.05

In addition, when 30 g/day of animal-based protein replaced by the same amount of plant-based protein, a significant decrease in BC risk in Model 2 was observed in all participants (Model 2: HR: 0.72; 95% CI: 0.59, 0.88) and women (Model 2: HR: 0.56; 95% CI: 0.37, 0.85). All significant associations disappeared in the final adjusted model (Table 5).

In dose-response analysis, no non-linear association was observed for animal protein replacement by plant protein (Supplementary Fig. 1).

### Sensitivity analysis

After omitting BC cases diagnosed within the first two years of follow-up, all the associations remained unchanged (Supplementary Tables 2–5).

## Discussion

In the present large prospective study, no significant associations were found between the intake of total protein animal-based protein, or plant-based protein and the risk of BC. Additionally, substituting plant-based protein with animal-based protein or vice versa did not show significant changes in BC risk.

To provide context, nationally representative data indicate that the average total protein intake in the general population is higher than that observed in our study (38.1 ± 17.4 g/day). For example, recent surveys suggest that average total protein intakes approximately range from 56.9 g/day to 85.8 g/day [51–54], highlighting that our sample may reflect a lower protein intake than the general population. Understanding these benchmarks is crucial for interpreting the implications of our findings.

The Health Professionals Follow-Up Study on 47,909 men showed that energy-adjusted total protein and animal protein intakes were not associated with the incidence of BC [55]. Another prospective study of 469,339 men and women and over 1,400 incident cases of urothelial cell carcinoma found no significant evidence that intake of energy from total protein is associated with the risk of urothelial cell carcinoma [56]; however, a 3% increase in the consumption of energy intake from animal protein was associated with a 15% higher risk of urothelial cell carcinoma while a 2% increase in energy from plant protein intake was associated with a 23% lower risk of urothelial cell carcinoma [56]. Bruemmer et al. [57] in a case-control study found no significant association between total protein intake and BC odds after adjusting for age, sex, country of birth, smoking status, and energy intake among middle-aged men and women. Moreover, some previous case-control studies found no association between total protein intake and BC risk [58, 59]. However, a US-based case-control study in men observed an inverse association between the highest and lowest categories of total protein intake, which was limited to a subgroup of older men [60].

Although it is difficult to separate the effects of high animal protein intake from low plant protein intake, there has been some hypothesis about the role of animal-based foods associated with the risk of BC, while the epidemiological findings are inconsistent. Evidence showed that animal protein may increase the concentrations of insulin-like growth factor-1, a peptide hormone associated with an increased risk of cancers, including BC [61, 62]. In addition, this increased risk might be due to the heme iron from red meat [24, 63, 64]. Heme catalyzes the formation of N-nitroso compounds and lipid oxidation end products, thereby potentially inducing carcinogenesis. In addition, heterocyclic amines (HCAs) and polycyclic aromatic hydrocarbons (PAHs), formed during high-temperature meat preparation, are suggested to increase genetic instability and to be carcinogenic via interaction with DNA [65]. Furthermore, HCAs are shown to be responsible for the mutagenic activity of cooked meats [24, 66]. On the other hand, although the effect of consuming plant-based proteins on BC and its mechanisms are still unknown, there is evidence that some plant-based components, such as isothiocyanates in cruciferous vegetables, flavones, and isoflavones are involved in pathways of inflammatory response, proliferation, and apoptosis of cells [67–69], thereby potentially playing an essential beneficial role in the prevention of BC in humans [9, 69, 70].

In contextualizing our findings, it is important to note that the average total protein intake in the general European adult population typically ranges from 67 to 114 g/day for adult men and 59 to 102 g/day for women, with dietary sources reflecting a mixture of animal and plant origins [71]. This is significantly higher than the total protein intake measured in the present study (38.1 ± 17.4 g/day). This suggests that the participants in our cohort may have had lower protein intake compared to the general population, which could influence the generalizability of our results.

In our study, adjusting for smoking status and alcohol intake in the final model led to the disappearance of significant associations. However, the observed associations between protein intake and BC risk may still be influenced by residual confounding from smoking, a known strong risk factor for BC [72]. Moreover, while animal-based protein intake is associated with body mass index (BMI) [73], we were unable to adjust for BMI due to insufficient data. It is also possible that unmeasured factors, such as specific dietary micronutrients, contributed to the associations in our study. However, there is no consistent evidence linking these factors to BC risk [74, 75], and any associations are unlikely to fully account for our findings.

To our knowledge, this is the first large prospective cohort study to evaluate the effect of animal-based protein and plant-based proteins and their substitution for one another on BC risk. In cohort studies on diet and cancer risk, the possibility of reversed causality can be a concern. Since removal of early diagnosed BC cases did not alter the results, we can conclude there is likely no reversed causality. Participants who got an BC diagnosis within a short period of follow-up, where therefore not excluded from the present study. However, the present study has several limitations. One notable limitation of our study is the absence of data on other cancer types within the cohort. As a result, we were unable to account for the potential confounding effects of prevalent cases of other cancers, which may have influenced BC risk. Future studies with comprehensive cancer registries could help address this gap and provide more robust insights into the relationship between dietary intake and BC. In addition, limited data was available on many possible confounding factors (e.g. BMI, physical activity, socioeconomic status, and exposure to carcinogenic chemicals). These variables are known to impact both dietary behaviors and cancer risk, which may introduce residual confounding into our risk estimates and the possibility of adjusting for these factors could provide more accurate risk estimates. Secondly, it is demonstrated that the cooking methods of foods may considerably affect the link between food consumption and BC risk. However, no data on cooking methods was available. Thirdly, measurement error and misclassification could have occurred using FFQs for the diet. As a result, information bias (a common bias in nutritional studies), as a consequence of self-reported information on dietary intakes, is to be considered when interpreting the results of the current study. Finally, the majority of the current study’s participants were of Caucasian ethnicity, which may limit the generalizability of the results to other ethnicities.

## Conclusion

In conclusion, the results of this large prospective study suggest that proteins from animal foods are positively associated with the risk of BC among current smokers. The intake of plant-based proteins, as a substitute for animal-based proteins, was associated with a lower risk of BC among current smokers. More studies are required to provide clear insights into the possible mechanisms of the effects of animal and plant-based proteins and the risk of BC.

## Electronic supplementary material

Below is the link to the electronic supplementary material.

Supplementary Material 1

## Data Availability

Datasets that are minimally required to replicate the outcomes of the study will be made available upon reasonable request.
