# Supplementary material for: The association between animal protein, plant protein, and their substitution with bladder cancer risk: a pooled analysis of 10 cohort studies
Source: Eur J Nutr. 2024 Dec 24;64(1):55. doi: 10.1007/s00394-024-03551-3 (PMC11668844; doi:10.1007/s00394-024-03551-3)
Supplement: Supplementary file 1 — Supplementary Material 1 [file 394_2024_3551_MOESM1_ESM.docx]

**Supplementary materials**

| Cohorts | VITAL | EPIC-Denmark | EPIC-France | EPIC-Germany | EPIC-Italy | EPIC-Spain | EPIC-Sweden | EPIC- the Netherlands | EPIC- the UK | EPIC-Norway |
| --- | --- | --- | --- | --- | --- | --- | --- | --- | --- | --- |
|  | N=31,809 | N=38,026 | N=59,843 | N=42,695 | N=37,067 | N=32,984 | N=65,475 | N=35,667 | N= 59,236 | N=31,610 |
| Participants (number) |  |  |  |  |  |  |  |  |  |  |
| Case | 178 | 238 | 26 | 169 | 165 | 131 | 229 | 104 | 177 | 23 |
| Cases, %* | 12.36 | 16.52 | 1.81 | 11.74 | 11.46 | 9.09 | 15.90 | 7.22 | 12.30 | 1.60 |
| Non-case | 31,631 | 37,788 | 59,817 | 42,526 | 36,902 | 32,853 | 65,246 | 35,563 | 59,059 | 31,587 |
| Abbreviations: EPIC: European Prospective Investigation into Cancer; VITAL: VITamins and Lifestyle study  * Percentage contribution of cases from each cohort | | | | | | | | | | |

**Supplementary Table 1.** Additional baseline characteristic for included cohort studies in BLEND study.

**Supplementary Table 2.** The association between dietary intakes of total proteins and bladder cancer after removing participants whose cancer was diagnosed in the first two years of follow-up

|  | Linear association (per 30 g) | Nonlinear association | | | |
| --- | --- | --- | --- | --- | --- |
|  |  | Tertile 1  < 28.4 g/day | Tertile 2  28.4 - 40.9 g/day | Tertile 3  > 40.9 g/day | P trend |
|  | HR (95% CI) | HR (95% CI) | HR (95% CI) | HR (95% CI) |  |
| **All participants** (cases/non-cases) | 1,256/432,972 | 405/144,345 | 403/144,328 | 448/144,299 |  |
| Person-year | 4,224,643.8 | 1,324,858.2 | 1,395,118.8 | 1,504,666.8 |  |
| Model 1 | 0.96 (0.84, 1.11) | 1 (ref.) | 1.07 (0.93, 1.24) | 1.14 (0.97, 1.35) | 0.09 |
| Model 2 | 0.98 (0.84, 1.13) | 1 (ref.) | 1.00 (0.85, 1.15) | 1.02 (0.86, 1.27) | 0.95 |
| Model 3 | 0.99 (0.84, 1.17) | 1 (ref.) | 1.12 (0.86, 1.18) | 1.03 (0.86, 1.24) | 0.88 |
| **Based on sex** (P for interaction>0.05) | |  |  |  |  |
| **Men** (cases/non-cases) | 845/115,083 | 241/31,202 | 267/37,695 | 337/46,186 |  |
| Model 1 | 1.20 (1.00, 1.43) | 1 (ref.) | 1.07 (0.91, 1.26) | 1.26 (0.88, 1.26) | 0.05 |
| Model 2 | 1.09 (0.96, 1.39) | 1 (ref.) | 1.06 (0.90, 1.26) | 1.11 (0.92, 1.41) | 0.05 |
| Model 3 | 1.12 (0.92, 1.36) | 1 (ref.) | 1.04 (0.87, 1.25) | 1.16 (0.93, 1.44) | 0.43 |
| **Women** (cases/non-cases) | 411/317,889 | 164/113,143 | 136/106,633 | 111/98,113 |  |
| Model 1 | 0.57 (0.44, 0.75) | 1 (ref.) | 0.89 (0.70, 1.14) | 0.61 (0.44, 0.84) | <0.01 |
| Model 2 | 0.62 (0.41, 0.79) | 1 (ref.) | 0.97 (0.77, 1.21) | 0.65 (0.44, 0.88) | 0.01 |
| Model 3 | 0.76 (0.56, 1.04) | 1 (ref.) | 0.93 (0.72, 1.20) | 0.78 (0.55, 1.10) | 0.21 |

HR, hazard ratio; CI, confidence interval

Model 1: Adjusted for total energy intake

Model 2: Additionally, adjusted for age and sex

Model 3: Additionally, adjusted for smoking status and alcohol intake

**Supplementary Table 3.** The association between dietary intakes of animal-based proteins and bladder cancer after removing participants whose cancer was diagnosed in the first two years of follow-up

|  | Linear association  (per 30 g) | Nonlinear association (per 30 g) | | | |
| --- | --- | --- | --- | --- | --- |
|  |  | Tertile 1  < 6.5 g/day | Tertile 2  6.5 – 15.1 g/day | Tertile 3  > 15.1 g/day | P trend |
|  | HR (95% CI) | HR (95% CI) | HR (95% CI) | HR (95% CI) |  |
| **All participants** (cases/non-cases) | 1,256/432,972 | 318/144,442 | 483/144,254 | 455/144,276 |  |
| Person-year | 4,224,643.8 | 1,544,886.6 | 1,424,251.8 | 1,255,505.4 |  |
| Model 1 | 1.52 (1.28, 1.80) | 1 (ref.) | 1.31 (1.13, 1.51) | 1.34 (1.15, 1.57) | <0.001 |
| Model 2 | 1.16 (1.02, 1.48) | 1 (ref.) | 1.19 (1.05, 1.41) | 1.12 (0.96, 1.35) | 0.06 |
| Model 3 | 1.08 (0.87, 1.35) | 1 (ref.) | 1.15 (0.99, 1.33) | 1.06 (0.89, 1.26) | 0.44 |
| **Based on sex** (P for interaction>0.05) | | | | |  |
| **Men** (cases/non-cases) | 845/115,083 | 201/33,814 | 312/38,394 | 332/42,875 |  |
| Model 1 | 1.52 (0.94, 1.40) | 1 (ref.) | 1.20 (1.01, 1.44) | 1.15 (0.95, 1.44) | 0.04 |
| Model 2 | 1.21 (1.02, 1.51) | 1 (ref.) | 1.19 (1.00, 1.39) | 1.20 (1.00, 1.40) | 0.05 |
| Model 3 | 1.14 (0.89, 1.46) | 1 (ref.) | 1.11 (0.92, 1.34) | 1.10 (0.89, 1.35) | 0.37 |
| **Women** (cases/non-cases) | 411/317,889 | 117/110,628 | 171/105,860 | 123/101,401 |  |
| Model 1 | 1.03 (0.71, 1.50) | 1 (ref.) | 1.25 (0.98, 1.60) | 0.96 (0.71, 1.28) | 0.77 |
| Model 2 | 1.12 (0.80, 1.66) | 1 (ref.) | 1.23 (0.97, 1.59) | 0.98 (0.73, 1.32) | 0.83 |
| Model 3 | 0.91 (0.57, 1.45) | 1 (ref.) | 1.21 (0.94, 1.56) | 0.92 (0.66, 1.29) | 0.99 |

HR, hazard ratio; CI, confidence interval

Model 1: Adjusted for total energy intake

Model 2: Additionally, adjusted for age and sex

Model 3: Additionally, adjusted for smoking status and alcohol intake

**Supplementary Table 4.** The association between dietary intakes of plant-based proteins and bladder cancer after removing participants whose cancer was diagnosed in the first two years of follow-up

|  | Linear association  (per 30 g) | Nonlinear association (per 30 g) | | | |
| --- | --- | --- | --- | --- | --- |
|  |  | Tertile1  < 17.8 g/day | Tertile2  17.8 – 27.9 g/day | Tertile3  > 27.9 g/day | P trend |
|  | HR (95% CI) | HR (95% CI) | HR (95% CI) | HR (95% CI) |  |
| **All participants** (cases/non-cases) | 1,440/432,972 | 461/144,263 | 432/144,309 | 363/144,400 |  |
| Person-year | 4224838.7 | 1087839.3 | 1569512.2 | 1567487.2 |  |
| Model 1 | 0.72 (0.62, 0.83) | 1 (ref.) | 0.93 (0.81, 1.07) | 0.82 (0.70, 0.96) | <0.001 |
| Model 2 | 0.70 (0.61, 0.90) | 1 (ref.) | 1.01 (0.89, 1.18) | 0.83 (0.71, 0.94) | 0.007 |
| Model 3 | 0.92 (0.77, 1.10) | 1 (ref.) | 1.01 (0.87, 1.18) | 0.94 (0.79, 1.12) | 0.21 |
| **Based on sex** (P for interaction>0.05) | | | | | |
| **Men** (cases/non-cases) | 845/115,083 | 297/35,654 | 280/36,565 | 268/42,864 |  |
| Model 1 | 1.08 (0.89, 1.30) | 1 (ref.) | 1.10 (0.93, 1.31 | 1.04 (0.86, 1.25) | 0.41 |
| Model 2 | 0.95 (0.76, 1.13) | 1 (ref.) | 1.02 (0.90, 1.23) | 0.92 (0.79, 1.12) | 0.36 |
| Model 3 | 1.01 (0.81, 1.25) | 1 (ref.) | 1.03 (0.85, 1.24) | 0.97 (0.79, 1.19) | 0.36 |
| **Women** (cases/non-cases) | 411/317,889 | 164/108,609 | 152/107,744 | 95/101,536 |  |
| Model 1 | 0.52 (0.39, 0.69) | 1 (ref.) | 0.94 (0.74, 1.20) | 0.62 (0.46, 0.83) | <0.001 |
| Model 2 | 0.50 (0.38, 0.66) | 1 (ref.) | 0.94 (0.74, 1.21) | 0.59 (0.45, 0.77) | 0.01 |
| Model 3 | 0.76 (054, 1.07) | 1 (ref.) | 0.99 (0.76, 1.28) | 0.89 (0.65, 1.22) | 0.43 |

HR, hazard ratio; CI, confidence interval

Model 1: Adjusted for total energy intake

Model 2: Additionally, adjusted for age and sex

Model 3: Additionally, adjusted for smoking status and alcohol intake

**Supplementary Table 5.** Substitution models where 30 g/day of dietary protein sources with each other after removing participants whose bladder cancer was diagnosed in the first two years of follow-up.

|  | | Animal-based protein for plant-based protein (30 g/day) | | | Plant-based protein for animal-based protein (30 g/day) | | |
| --- | --- | --- | --- | --- | --- | --- | --- |
|  | | Model 1  HR (95% CI) | Model 2  HR (95% CI) | Model 3  HR (95% CI) | Model 1  HR (95% CI) | Model 2  HR (95% CI) | Model 3  HR (95% CI) |
| **All participants** | | 1.71 (1.40, 2.08) | 1.34 (1.09, 1.54) | 1.11 (0.87, 1.43) | 0.58 (0.47, 0.71) | 0.71 (0.52, 0.94) | 0.89 (0.69, 1,14) |
| **Based on sex*** | **Men** | 1.01 (0.80, 1.28) | 1.17 (1.06, 1.56) | 1.08 (0.81, 1.43) | 0.98 (0.77, 1.24) | 0.96 (0.71, 1.01) | 0.92 (0.69, 1.22) |
|  | **Women** | 1.83 (1.21, 2.76) | 1.78 (1.18, 2.70) | 1.11 (0.65, 1.87) | 0.54 (0.36, 0.82) | 0.49 (0.31, 0.81) | 0.90 (0.53, 1.52) |

HR, hazard ratio; CI, confidence interval

Model 1: Adjusted for total energy intake

Model 2: Additionally, adjusted for age and sex

Model 3: Additionally, adjusted for smoking status and alcohol intake

*P heterogeneity>0.05


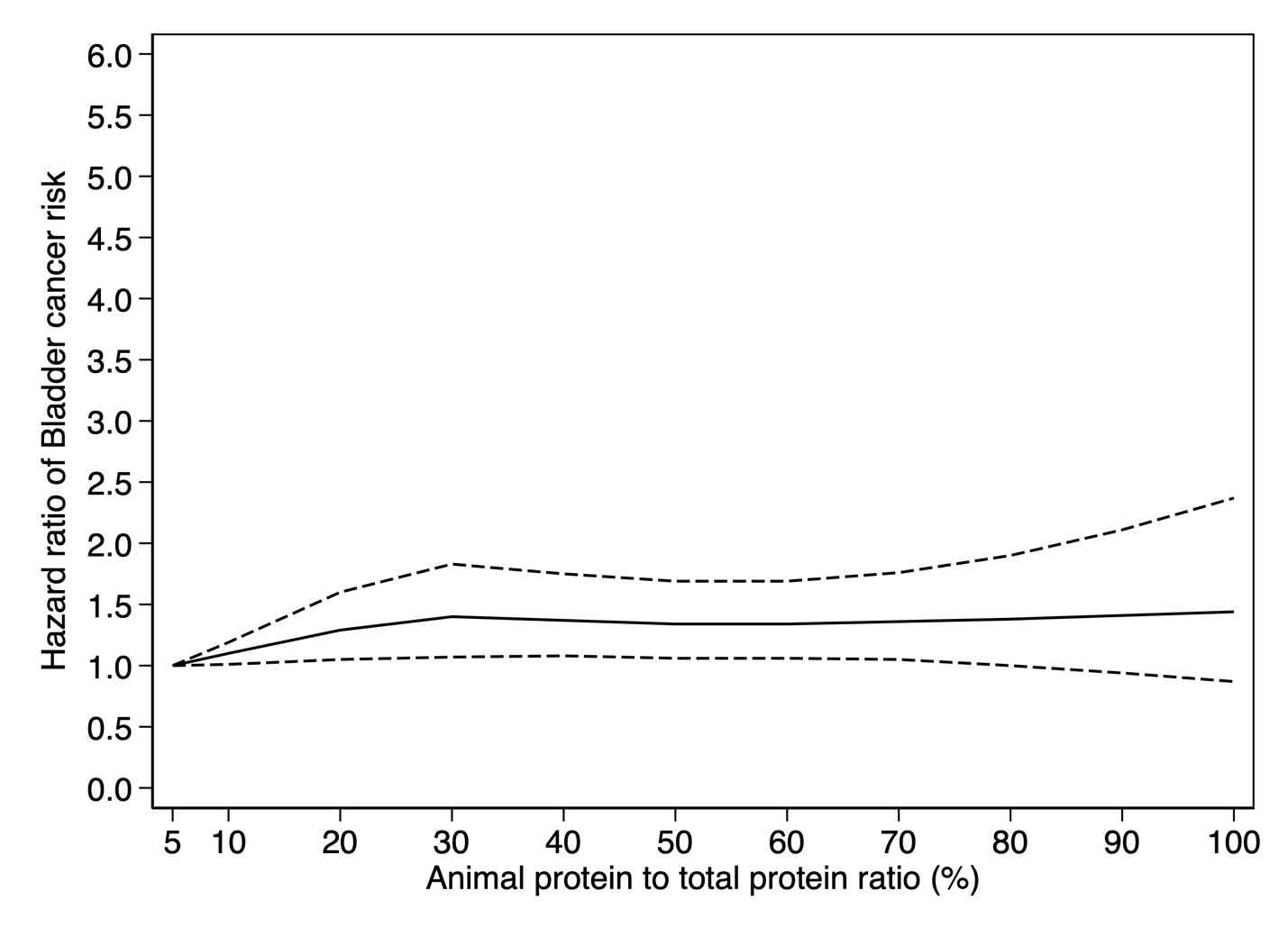


**Supplementary Figure 1.** Relative risk of BC for substituting of animal protein for plant protein. Lines are restricted cubic splines presenting the shape of dose–response curve based on animal protein to total protein ratio on a continuous basis: relative risks (—); 95% CI (– – –). The reference point is lower than 5% animal protein to total protein ratio with four knots. All models were adjusted for total energy intake, age, sex, smoking status, and alcohol intake.
